# From Svalbard to Siberia: Passerines breeding in the High Arctic also endure the extreme cold of the Western Steppe

**DOI:** 10.1371/journal.pone.0202114

**Published:** 2018-09-05

**Authors:** Katherine R. S. Snell, Bård G. Stokke, Arne Moksnes, Kasper Thorup, Frode Fossøy

**Affiliations:** 1 Center for Macroecology, Evolution and Climate, Natural History Museum of Denmark, University of Copenhagen, Universitetsparken 15, Copenhagen, Denmark; 2 Norwegian University of Science and Technology (NTNU), Department of Biology, Høgskoleringen 5, Trondheim, Norway; 3 Norwegian Institute for Nature Research (NINA), Torgarden, Trondheim, Norway; Fred Hutchinson Cancer Research Center, UNITED STATES

## Abstract

Few species are adapted to high latitudes, and many over-winter in milder climates with migrations involving extensive barrier crossings. By escaping extreme conditions for the majority of the year, physiological and behavioural adaptations presumably need to be less pronounced. The snow bunting *Plectrophenax nivalis* is the most northerly breeding passerine. We tracked the Svalbard population using geolocators to reveal that these individuals not only breed in environmental extremes, but also spend the winters in the severe cold and highly stochastic weather conditions of the Siberian steppe. Migratory strategies appeared to be flexible between individuals and years. However, common wintering grounds in the Asian Western Steppe were identified, where birds could utilise vast crop- and grasslands while enduring low ambient temperatures. The timing of significant long distance movements was consistent among individuals, and the autumn routing of the >1000 km open water flight to Novaya Zemlya incurred favourable wind assistance and lower risk of precipitation, compared to the shorter route between Svalbard and Norway used in spring. Presumably, Svalbard snow buntings are physiologically well-adapted to extreme conditions and their migration, rather being a retreat from physiologically demanding conditions, allows utilisation of an abundance of resources in the Asian Steppe.

## Introduction

Bird migration operates on a vast scale: birds are known to travel enormous distances and negotiate inhospitable geographical features such as oceans, mountain ranges and deserts, presumably to exploit seasonal resources [1]. While the number of bird species decreases with latitude, the proportion of migrants increases [2]. Polar species are largely obligate migrants; the polar summer provides favourable conditions for breeding (long daylight, high plant and invertebrate production and greater stability of weather patterns) whereas winter conditions (snow cover, darkness, low production and frigid temperatures) are not conducive to endotherms [3]. As a result, high latitude species demonstrate inherent abilities to tolerate environmental extremes, and have adapted physiologically and behaviourally to the variation in climate and habitats and to long-distance travel [2, 4].

Long-distance migrants generally spend the non-breeding period in distinctively milder climates such as the Tropics. Intra- and inter-continental flights invariably involve negotiating ecological barriers, with limited landing or foraging opportunities. Most ecological barriers are not physically restrictive to flight, nevertheless, in combination with adverse and unpredictable winds and weather events, they are expected to influence the development of migratory routes and timings [5].

Snow buntings nest at higher latitudes than any other passerine, from 50.1° to 83.6° North [6, 7]. Their breeding range is circumpolar with most of the non-breeding range outside the polar region. A number of populations are separated by large-scale ecological barriers [8] and migratory routes vary. The breeding range is highly dispersed and migratory connectivity is largely unknown, although the major migration routes in Northern Europe and the New World have been inferred by ring recoveries [7, 9, 10].

Here, we focus on a population of 1,000–10,000 pairs of snow buntings breeding on Svalbard [11], the Norwegian sovereign archipelago in the Arctic Ocean. These birds are present from late April until early September but spend the majority of the year away from their breeding grounds, as also recorded in North American birds [10]. The wintering grounds of the Svalbard population are unknown (one autumn and a few spring recoveries in Arkhangelsk; a few recovered in northern Norway in spring [12]). However ring recoveries for the western palearctic suggest three potential routes: (i) to continental Europe joining northern Scandinavian birds, (ii) eastwards to the White Sea before turning southward or (iii) westwards traversing Greenland to winter in the New World [13–17]. All potential routes from Svalbard incur an extended water crossing.

Identifying the spatiotemporal schedules of small migrants has only recently become possible with the miniaturisation of light-level geolocators [18]. These archival loggers have low spatial resolution compared to GPS or satellite tags and are compromised at high latitudes by continuous daylight; however, they are applicable for determining movements, timings and broad scale spatial ranges.

In contrast to the Canadian populations [10], birds from Svalbard must navigate across an ecological barrier, the cost of which is presumably offset by the arrival at more benign winter grounds. Hence we fitted breeding snow buntings with geolocators to map the spatiotemporal annual cycle of the Svalbard High Arctic population and to allow us to investigate potential advantages and disadvantages of their obligate migration. The tracks revealed that birds wintered on the Siberian Steppe. Satellite derived environmental data were used to quantify the environmental conditions both at population scale utilisation sites in the non-breeding period and those experienced at the individual level, in order to identify wintering conditions and to describe habitat requirements for the greatest proportion of the annual cycle. We used weather and environmental variables to further investigate route selection on flight performance during the ocean crossing.

## Materials and methods

### Permits

Permissions to carry out the study were given by The Governor of Svalbard (2014/00375-5), The Norwegian Animal Research Authority (FOTS ID 4701) and the landowner, Store Norske Spitsbergen Grubekompani. The snow bunting project in Adventdalen is registered as RiS-ID 2272 in the Research in Svalbard Database.

### Geolocators

A total of 41 snow buntings breeding in nest boxes near Longyearbyen, Svalbard (78.2°N 15.8°W) part of an on-going long-term project [19], were fitted with light-level geolocators (Intigeo P65C2-7, Migrate Technology, UK; 0.74 g; leg-loop harness of 1 mm nylon cord) during 2014 and 2015. Geolocators were retrieved in the following summers from 12 birds (10 males and 2 females; and included two individuals with repeated tracks). Return rates of birds with colour rings between 1999 and 2003 were comparable (28.3–43.1% [20]).

Light data were corrected for clock drift. Positions were calculated from the threshold method using the GeoLight package [21] in R [22] and sun elevation angles were calculated from breeding site calibration during a period of known twilight events prior to autumn departure. Reference geolocators were deployed at the breeding site to determine onset of twilight detections following polar summer. As this period is typically short and in general the return to the colony is obscured by polar day, latitudinal calibration was verified using Hill-Ekstrom calibration and the FlightR package [23]; and confirmed that tracks were overall similar and stationary periods were within the latitudinal range used to identify significant movements. Due to the equinox effect on position estimation, latitudes 14 days before and after equinoxes were excluded. Key phases of the migration, which involved extended directed flight, were identified from these positions: ocean crossing departing from Svalbard; arrival at wintering grounds; arrival at spring staging; ocean crossing departing to Svalbard. The intervals were classed as ‘autumn’, ‘winter’ and ‘spring’. Major spatial use areas for each seasonal subset were estimated by applying kernel density (KDE) analysis to positions (adehabitatHR R package [24]). KDEs partially account for imprecision in light-derived positions [25]. Stationary periods were assigned to consecutive positions with less than 2° variation in the latitude and longitude for durations of 5 days or more. For stationary periods, the median latitude and longitude were used in further analysis. Significant movements were defined as changes of greater than 5° per twilight interval between stationary periods. Raw light-level data files are available from www.movebank.org; ID 540289187.

### Environmental conditions and land use

Surface temperature, snow depth, u- and v-winds (geographic wind coordinate system), precipitable water, vertical wind shear and ice cover were obtained from NCEP FNL re-analysis product [26]. Daily conditions associated with individual tracks were interpolated for NCEP data for stationary periods using the rNCEP package [27, 28]. Actual air temperature in the wintering range was derived from all 7 permanent weather stations at airfields within the 70% KDE of all positions (UAUU, USCC, UWUU, USCM, UWWW, UWOO and UWOR [29]). Longyearbyen airport (ENSB) data was used for temperature and precipitation at the breeding site. Mean values were calculated from daily summary statistics for periods of interest. Snow and ice cover were verified from cloud free EOSDIS worldview composite satellite images with Terra/MODIS sea ice layer and snow cover [30]. Land cover taken from IGBP classifications (water, evergreen needleleaf forest, deciduous broadleaf forest, mixed forest, open shrubland, woody savannas, grasslands, croplands, urban and built-up, cropland/natural vegetation mosaic were encountered in our area of interest), and percentage crop cover were derived from MODIS MOD12C1 0.25 Degree Land Cover Collection [31]; data accessed using the ORNL DAAC Spatial Data Access Tool [32].

### Ocean crossing conditions

The autumn route used between Svalbard and continental Europe was longer than the alternative available and predominantly used in spring. We tested the environmental conditions encountered over the two routes on the respective dates of autumn migration by individuals. The routes crossing the Barents Sea were defined as (i) the actual: a great circle route between the breeding site and closest landfall on Novaya Zemlya within the autumn 70% KDE (74.4°N 55.3°E, 1096 km) and (ii) an alternate known route: the reciprocal spring great circle route from northern Scandinavia within spring 70% KDE (71.1°N 28.1°E; 864 km). The tailwind component, precipitable water and vertical wind shear for the entire route for 24 hours post departure were tested with two-tailed sign tests. The tailwind component was calculated from NCEP mean wind vectors at 925 mbar with 12 m s^-1^ flight speed [27, 28] for each bird on their chosen departure date, for each of the two routes.

## Results

### Wintering

**Fig 1:**
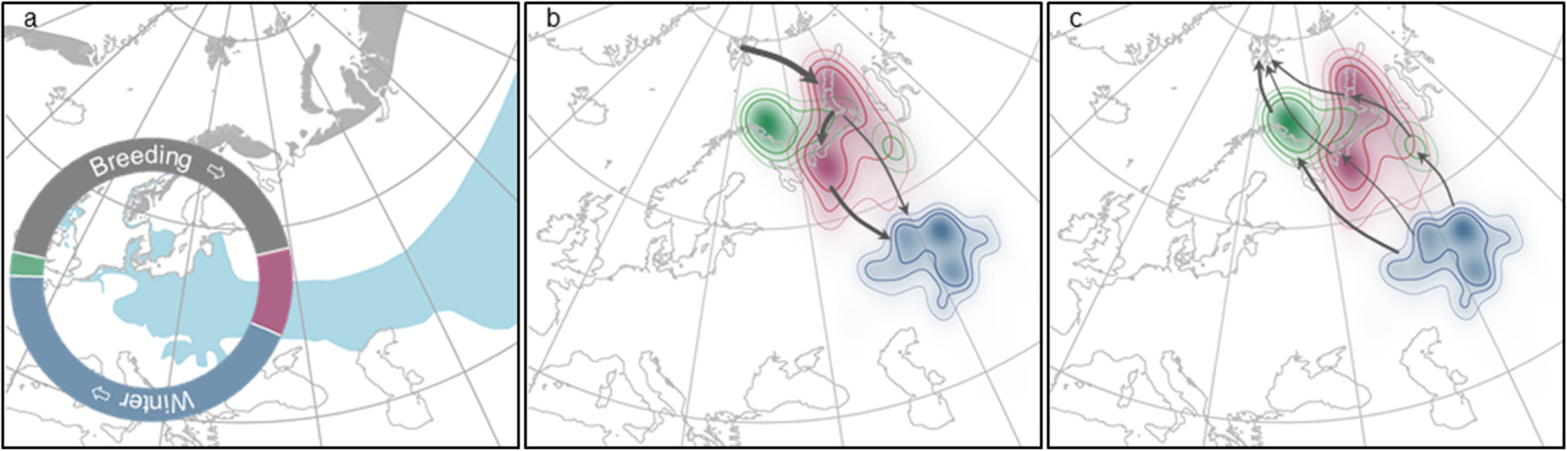
Map of snow bunting distribution ranges, time budgets and migration routes and stopovers of tracked birds. (A) Map of breeding (grey) and wintering (blue) distribution of snow buntings from Birdlife International [6], and mean annual time budget from tracked birds (autumn migration (magenta) and spring migration (green); orientated with 1 June uppermost, n = 12). Maps of position estimates for tracked birds and kernel density estimation (KDE) for each temporal period (contours at 30, 50 and 70% KDE, n = 12); and routes (indicated by proportionate arrows) undertaken for autumn (B) and spring (C) migration.

All birds wintered in the Western Steppe region in Siberia (n = 12). The wintering period constituting the largest portion of the annual cycle (44%; median duration 161 days, range 154–173 days; Fig 1, S1 Table). The area used was vast, ranging from 43.7°-70.8°E and 47.5°-56.3°N (S1 Fig). Movement behaviour during the winter varied between individuals. A quarter of birds remained entirely stationary for the duration of the winter period; up to one individual with 4 distinct and directed within-winter migratory movements (S2 Fig). Eight of the 12 birds exhibited some degree of nomadic behaviour (from 3 to 100 days; S1 and S2 Figs, S1 Table).

**Fig 2:**
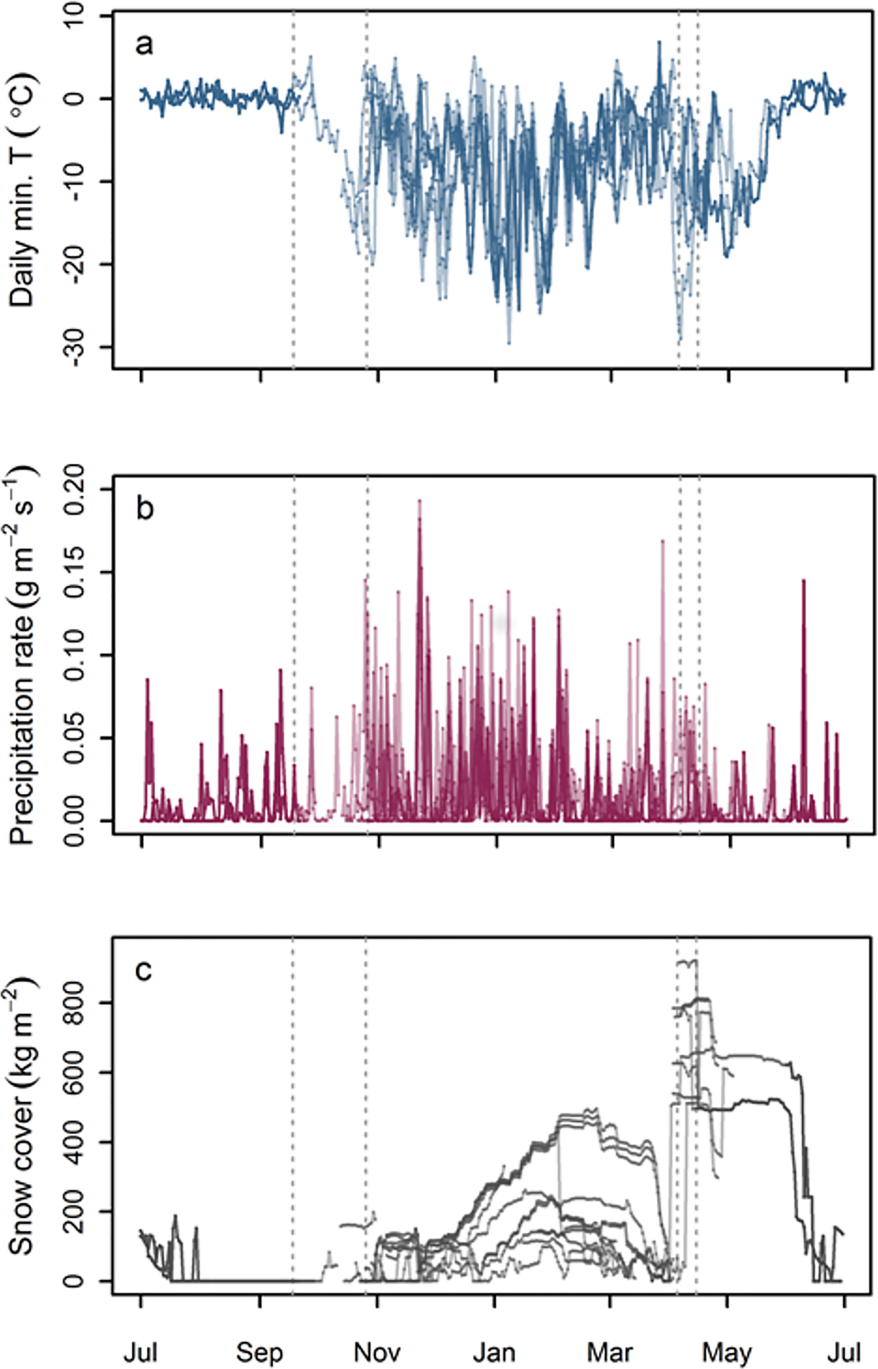
Environmental variables encountered by individuals. Environmental variables encountered by individuals (n = 12) throughout the annual cycle where stationary positions can be estimated from geolocator data: (A) daily minimum surface temperature (°C), (B) daily mean precipitation rate (g m^-2^s^-1^) and (C) snow cover as snow water equivalent (kg m^-2^). For illustration mean dates of breeding, autumn, winter and spring intervals are indicated by a dotted line.

The wintering grounds (S3 Fig) are dominated by vast croplands and grasslands where ambient winter temperatures are low. The absolute minimum NCEP-derived temperatures for each cell within the wintering site (area defined by the 70% KDE) ranged from -42.5° C to -24.8°C with mean temperatures ranging from -10.9°C to -3.6°C (Figure a & b in S3 Fig). Daily minimum surface temperature ranged between -31°C to 2°C and maximum temperature ranged between -22°C to 9°C. Daily temperatures (Fig 2A) associated with individual tracks ranged from -27.5°C to 7.7°C (mean) and -29.5°C to 6.8°C (minimum). Extremes were encountered during the wintering period, and were characterised by a high degree of stochasticity. Mean and minimum temperatures showed the same pattern. The wintering site was predominantly in areas of extensive croplands categorised as 57.6% croplands, 30.1% grasslands and 9.2% mixed forest (IGBP classification), and a median of 62.1% croplands percentage cover (Figure c in S3 Fig). The mean NCEP forecasted maximum snow depth was 0.63 m (range 0.13–0.95 m; Figure d in S3 Fig). However, the first lying snow for this area was on 23 January 2015; cloud cover precluded identification of first snows in the second season. Maximum snow cover and precipitation rates for individual tracks were 921 kg m^-2^ and 0.19 g m^-2^ s^-1^, respectively (Fig 2B and 2C), with the greatest number and magnitude of precipitation events occurring during the winter period resulting in accumulation of snow cover up to 492 kg m^-2^. However, the largest snow accumulation encountered was during the northward spring migration, and snow cover of up to 648 kg m^-2^ on return to Svalbard, which melted rapidly in mid-June.

### Migration routes and timing

The snow buntings left their breeding grounds in Svalbard (Fig 1) making landfall on either Novaya Zemlya Archepelago/Nemesia (n = 11) or the Eastern Kola Peninsula (n = 1). All autumn ocean crossings were directed towards the east or southeast, crossing between 25° and 51° longitude. Generally, the birds appeared to follow a clock-wise loop migration although routes varied considerably during the non-breeding period with 4 of the 12 tracks routing to the eastern side of the Ural Mountains while the majority moved westwards after making landfall. The few repeated tracks (n = 2) indicated that the same individuals took different routes in subsequent seasons. A number of discrete short stop-overs could be identified during the autumn migration while spring migration was relatively direct back to the breeding grounds with a stop-over in Northern Scandinavia (n = 6), via the Kanin Peninsula (n = 2) or apparently retracing the autumn migration route (n = 4).

Timing of significant movements showed a high degree of synchrony among individuals both in departure dates from Svalbard and movements within the autumn and spring migrations (S1 Table). The two female birds undertook the autumn Barents Sea crossing earlier than the males in both years (14 & 15 September). Males departed 16–21 September except one on 30 September. The birds arrived at their overwintering area in the Asian Steppe 11 October– 6 November where they remained until early April. Of the 12 tracks, only four birds remained stationary throughout the wintering period (S2 Fig, S1 Table). The remaining birds displayed nomadic behaviour for part of the winter, or relocated, resulting in multiple stationary periods within the Western Steppe (separated by up to c. 650 km). All birds flew north in spring within 18 days of each other, however 9 of the 12 birds began their spring migration from the wintering grounds during the narrow interval of 1–4 April. Where the return ocean crossing to Svalbard could be identified (before the onset of polar days), the earliest was from Novaya Zemlya on 6 April; four other departures were from the Kanin Peninsula (n = 1) on 13 April and Northern Scandinavia (n = 3) on 15 and 16 April.

**Fig 3:**
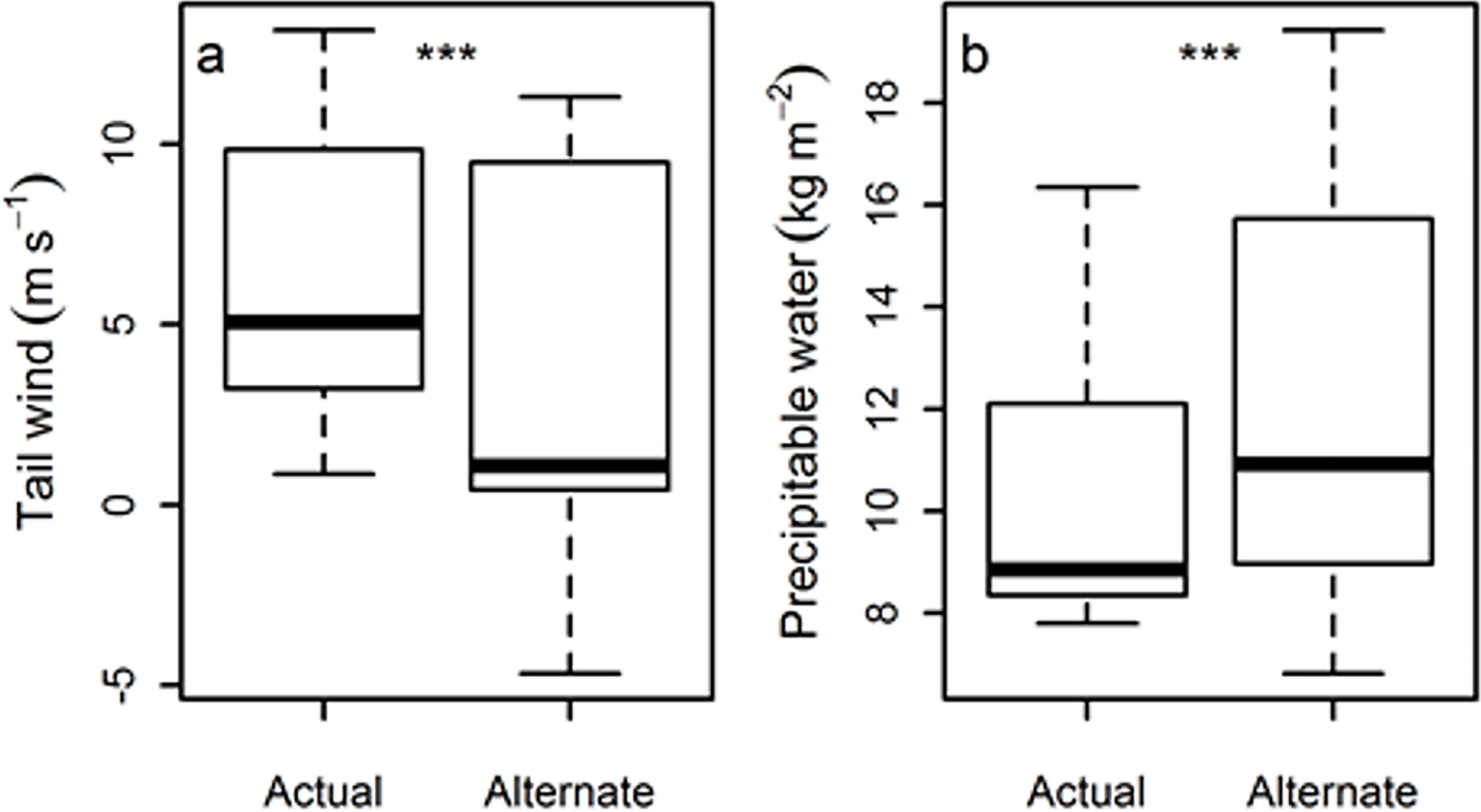
Environmental conditions. Environmental conditions encountered on actual autumn migration compared to the alternate shorter route used by some birds in spring: (A) Median tail wind component (m s^-1^) and (B) Precipitable water (kg m^-2^) of route from Svalbard to Novaya Zemlya and alternate route to Northern Scandinavia on known departure dates (n = 12).

The autumn ocean flight from Svalbard to Novaya Zemlya afforded significantly greater wind assistance and less precipitable water than the shorter reciprocal Svalbard to Northern Scandinavian route. The median tail-wind component for the chosen route ranged from 0.8 to 13.2 m s^-1^ whereas the alternate route investigated here experienced more headwinds, -4.7 to 11.3 m s^-1^ (mean of the differences = 3.04 m s^-1^, S = 10, *P* = 0.038; Fig 3A). The resultant calculated median flight times were 17.8 h and 18.3 h respectively. The median precipitable water ranged from 7.8 to 16.35 kg m^-2^ for the actual route compared to 6.81 to 19.43 kg m^-2^ for the alternate route (mean of differences -1.78 kg m^-2^, S = 2, *P* = 0.038; Fig 3B). No difference in vertical wind shear was found between routes (chosen route encountered potential vorticity range of between 0.0027 to 0.0110 km^2^kg^-1^s^-1^ whereas the alternate was 0.0015 to 0.0115 km^2^kg^-1^s^-1^). No sea ice was present on either route. Once landfall was made the route was not direct towards the wintering grounds, suggesting birds may be following key topographical features (rivers or mountain ranges).

## Discussion

Severe environmental conditions throughout an extensive area of the Western Steppe region were experienced by the migratory Svalbard snow buntings during the non-breeding period. Departure from Svalbard and the ocean ecological barrier crossing were temporally and spatially consistent between individuals and years. However, following landfall on the continent, a high degree of variation in routes and nomadic behaviour was recorded between individuals and years. The wintering range was associated with low ambient temperatures, crop- and grass-lands, indicating this population’s ability to endure extreme temperatures provided there is an abundance of resources. Spring migration was characterised by narrow temporal range but individualistic routings. The autumn barrier crossing of more than 1000 km—not by the shortest distance available—incurred favourable wind and precipitation conditions, which may explain this surprising route. Because we treat data conservatively, these conclusions are unlikely to be affected by the low precision and accuracy of the geolocator data, though tracking of the return over water flight to Svalbard was precluded.

### Wintering area conditions

Contrary to our understanding of arctic breeding species, considerably harsher environmental conditions were endured by all birds during the non-breeding period than was experienced in the high Arctic of their breeding grounds [2]. Migratory species are believed to escape seasonally inhospitable regions and endure extreme conditions only during essential life history periods e.g. breeding [3, 33]. However, these snow buntings spent the majority of their annual cycle on the Western Steppe where ambient air temperatures are low in absolute terms; moreover extensive stochasticity of conditions was encountered. Snowfall was greater during the non-breeding period and snow accumulation experienced by individuals varied considerably. Weather data indicated that generally birds encountered substantial snow cover from December onwards, which must be incorporated into their foraging strategies. Satellite imagery of the area, however, shows that the first lying snow cover on the 23 January in 2015 is consistent with high wind conditions scouring lying snow, leaving flat ground exposed until late winter [34]. Nevertheless, these arctic conditions necessitate specialisation for survival and are indicative that snow buntings are a highly adapted polar species [35].

Although the Western Steppe is a known wintering area for snow buntings [36] their origin was unknown. Our tracking data reveal that the Svalbard population utilise this region during the mobile and longest period of their annual cycle. Although their migration route in part overlaps with the Finnmark breeding range, the wintering regions of these two populations appear to be discrete [12]. The combination of relatively harsher conditions and the leap-frog migration strategy is indicative of a wintering niche. We propose that the most northerly breeding population overwinters in colder regions while more temperate breeders predominantly utilise coastal regions of Europe [7].

These birds may take advantage of foraging opportunities with little competition from conspecifics unable to withstand these challenging conditions. Wintering locations are consistent with birds foraging on spent grain from the vast croplands of the Western Steppe. Within this region, grasslands have been cultivated for over two centuries forming near-continuous large scale intensive agriculture [37], dominated by wheat production (96.3% of arable). In addition, tracked birds overwinter on natural grasslands which are likely to provide foraging opportunities similar to fallow croplands over winter. Few other ground-foraging passerine species are reported in the region over winter (Lapland bunting *Calcarius lapponicus* and twite *Linaria flavirostris*), indicative of the cold tolerance of snow buntings and the ecological niche strategy of reduced competition [38, 39].

### Route and timing

The consistency of migration timing throughout the annual cycle can be explained by intrinsic and extrinsic factors [3]. The observed decrease in temperature and precipitation events was concurrent with departures, and may therefore initiate southwards migration. Although not all birds depart on the same day, the consistencies in timing between years may be a consequence of the weather (escaping harsh conditions and taking advantage of tail winds) rather than an endogenous timing response.

Snow buntings take advantage of prevailing wind conditions to optimise autumn migration with reduced flight time and risk, despite incurring a substantially longer ground track across the Arctic Ocean. Our data revealed a surprising eastwards route selection from Svalbard. Birds undertook an overwater flight (minimum of 1096 km) considerably longer than the shortest distance from Svalbard to northern Scandinavia (640 km) and a known reciprocal spring route (minimum of 864 km). Local environmental conditions of greater wind assistance and less potential precipitation can explain this extended crossing. Tøttrup et al. [40] showed that red-back shrikes *Lanius collurio* take advantage of favourable wind conditions on autumn migration. However, this species also minimised the ground track as well as flight time over the environmental barrier. For our snow buntings, the calculated mean flight time of both known routes is similar, with an overall advantage for the observed autumn route despite the further ground distance. The eastwards route may reflect improved foraging conditions [41], provide a greater chance of making landfall after the water crossing in the event of crosswind deviation or be associated with more consistent and predictable (i.e. low risk) conditions in autumn [5]. While we did not conduct a comprehensive survey of all possible routes and timings available to the birds in order to make landfall in continental Europe, the advantages associated with the actual route are unlikely to be affected by choice of the alternate route tested. An alternative explanation for this initial eastward movement is the historic population recolonization after the last glacial maximum from Asia and birds are following inherent reciprocal dispersal routes.

The ocean crossing is still a considerable obligate ecological barrier for this breeding population [42]. Geolocator tracked snow buntings in Canada were shown to migrate in the order of 2100 km, approximately half that of the Svalbard population and without an ocean crossing stage [43]. However the protracted autumn migration route is clearly still within the capabilities of this species. Northern wheatears *Oenanthe oenanthe* migrating from Europe to Iceland encounter a similar transoceanic crossing (minimum ground distance of *c*. 860 km); however their flight endurance based on fat stores is calculated to be over 1450 km [44]. Moreover, this appears trivial compared to the apparent regular passerine migration directly across the Gulf of Mexico, where numerous species are thought to make around 1000–2550 km overwater flights with only scattered islands as refuges [45–47], including the 12 g Blackpoll warbler *Setophaga striata* with a described non-stop migration of 2270–2770 km [48].

In autumn, snow buntings leaving Svalbard take advantage of optimal flight conditions. However conserved timing of ocean crossings, from their breeding grounds to the continent, may also be explained by birds traveling as small flocks [49]. Males and female demonstrate timing differences concurrent with known protandry in this species [16, 43, 50], consistent with apparent sex-specific thermal tolerance (described by Macdonald et al. [43]).

Migration may have immense influence on fitness [51]. On spring migration, as birds rapidly move northwards, they encounter regions of extensive snow cover but reduced precipitation. The return route through northern Scandinavia has the advantage of a shorter over-water distance and, additionally, it may also allow sampling of environmental conditions for optimal timing of arrival. Specific functions of spring stopover have been previously suggested [10] and tracked Canadian birds spend considerably longer at stopover sites than during autumn migration [10, 52]. However, birds with known return dates (concurrent with observations in Svalbard [53]) are clearly arriving at the breeding grounds under suboptimal conditions, with deep snow cover and negative temperatures well into May, as reported in Greenland and Alaska [16, 54].

Timing of spring migration is anticipated to be indirectly linked to onset of breeding, and may be driven by conditions encountered during the northwards route. Phenology of breeding is governed primarily by photoperiod [55, 56] with further regulation by non-photic cues such as temperature [57]. Although high latitude species are recorded to be more resistant to temperature augmentation of photoperiod-induced gonadal growth, advancement of breeding schedule correlated to early spring temperature warming has been recorded for this species in Svalbard and Alaska [19, 57, 58]). The resultant mistiming from photoperiod and importantly arthropod availability was detrimental for nestling development and overall productivity, suggesting that phenology in this species is particularly sensitive to environmental conditions. Due to the onset of polar day, the arrival date of all tracked breeding birds cannot be determined. At present, it is unknown if timing of spring migration may contribute to the pattern of advanced commencement of breeding, and should be further investigated when technologies exist.

### Physiological adaptations

The Svalbard population of snow buntings is undoubtedly suited to extreme conditions, due to a suite of probable physiological and behavioural adaptations. Few small passerine species overwinter in arctic or sub-arctic conditions, and even populations of the same species in North America find improved conditions over the winter period than at their breeding sites [54].

Birds normally maintain their body temperature within narrow limits and are specialised to thermal habitats. Snow buntings can maintain their body temperature over a temperature range between -40°C and 40°C [4] and can withstand temperatures down to -50°C. Alaskan snow buntings only require compensatory metabolic increases below ambient temperatures of 10°C however Svalbard snow buntings rarely experience temperatures above this threshold. Physiological adaptations that may allow these birds to persist under extreme, energetically challenging, conditions which include seasonal acclimation to cold, through a complex suite of metabolic and morphological adjustments [59] such as insulative capacity from plumage, increased facultative hypothermic response [60–62] and increased cellular metabolic rate and maximum thermogenic capacity [63, 64].

Sustained high levels of heat production (cellular metabolism and supplementary thermogenesis), principally by pectoral skeletal muscle shivering, is positively correlated to muscle mass [65, 66]. Pectoral muscle in migratory birds is both the largest and most energy demanding organ [67]. The obligate endurance flight across the ecological barrier and wintering thermogenesis may promote the maintenance of increased muscle mass and is evidence of physiology shaping spatial migratory patterns.

The multiple strategies across individuals/seasons of a succession of stationary periods and nomadic periods are indicative of generalist behaviour. Such itinerancy has been demonstrated over relatively small distances in wintering snow buntings [10, 68], however, within-winter movements in other small songbirds ranging from willow warblers *Phylloscopus trochilus* to the common cuckoo *Cuculus canorus* are better described as multiple non-breeding sites rather than the complex nomadic behaviour we reveal here [69–72]. This highlights the need for better understanding of within-winter movements across taxa, both at the microhabitat tracking scale (resources or climate) to longer flights in response to extreme conditions [1, 73].

## Conclusion

We demonstrate that a high-arctic breeding passerine, the snow bunting, is specialised to extreme environmental conditions throughout its annual cycle. Extreme low temperatures and extensive snow cover are endured over winter and on arrival at the breeding grounds. The considerable obligate barrier crossing to escape worsening conditions in Svalbard is ameliorated by a route selection involving a longer ground track but with clear time and risk savings. Considerable physiological and behavioural adaptations are required under consistent arctic conditions and during extensive, obligatory, migratory flights.

## Supporting information

S1 Fig Migration of snow buntings. Migration of snow buntings from breeding grounds to breeding grounds (individuals represented by colour and logger ID). Identified stationary periods represented by solid line (—**-**) and periods of apparent movement by a dashed line (**----**), these include directed migration and nomadic behaviour. Breeding site longitude and latitude indicated by grey dotted line. Estimated positions represented by grey dots, and latitudes affected by the equinox are excluded.
(PDF)

S2 Fig Time series of snow buntings. Time series of identified migratory movements, stationary periods and nomadic behaviour. Key migration events are depicted by ▼ southwards and ▲ northwards, and coloured by departure from Svalbard, autumn (red), arrival at wintering grounds (blue), departure from wintering grounds, spring (green) and where known, departure from mainland to Svalbard (yellow). Additional movements within these periods are denoted by ◆. Stationary periods are indicated by black lines (━) and apparent nomadic behaviour by grey lines.
(PDF)

S3 Fig Wintering environmental variables and land use. Environmental variables and land use for the duration of the wintering periods, with 70% Kernel Density Estimation of wintering grounds depicted by black polygon: (a) mean daily mean surface temperature (°C), (b) absolute minimum surface temperature (°C), (c) Percentage crop cover and (d) maximum snow depth (m), note that snow cover over oceans indicates maximum extent of sea ice.
(PDF)

S1 Table Tracked birds deployment information and key timings. Tracked snow bunting deployment information and key timings of significant migration which were used to define Autumn, Winter and Spring periods. Total number of stationary periods (SP) and duration (in days) of wintering stationary periods, defined Autumn and Winter periods, and where known Spring and Breeding grounds for the 12 month period the birds were tracked. Summary statistics, where appropriate are included for day of migration and duration of periods, however the period of spring and breeding are under- and over-estimated due to lack of return dates for 7 birds due to the effect of Polar day.
(PDF)
