# Supplementary material for: From Svalbard to Siberia: Passerines breeding in the High Arctic also endure the extreme cold of the Western Steppe
Source: PLoS One. 2018 Sep 5;13(9):e0202114. doi: 10.1371/journal.pone.0202114 (PMC6124700; doi:10.1371/journal.pone.0202114)
Supplement: S1 Fig — Migration of snow buntings from breeding grounds to breeding grounds (individuals represented by colour and logger ID). Identified stationary periods represented by solid line (—-) and periods of apparent movement by a dashed line (----), these include directed migration and nomadic behaviour. Breeding site longitude and latitude indicated by grey dotted line. Estimated positions represented by grey dots, and latitudes affected by the equinox are excluded. (PDF) [file pone.0202114.s001.pdf]

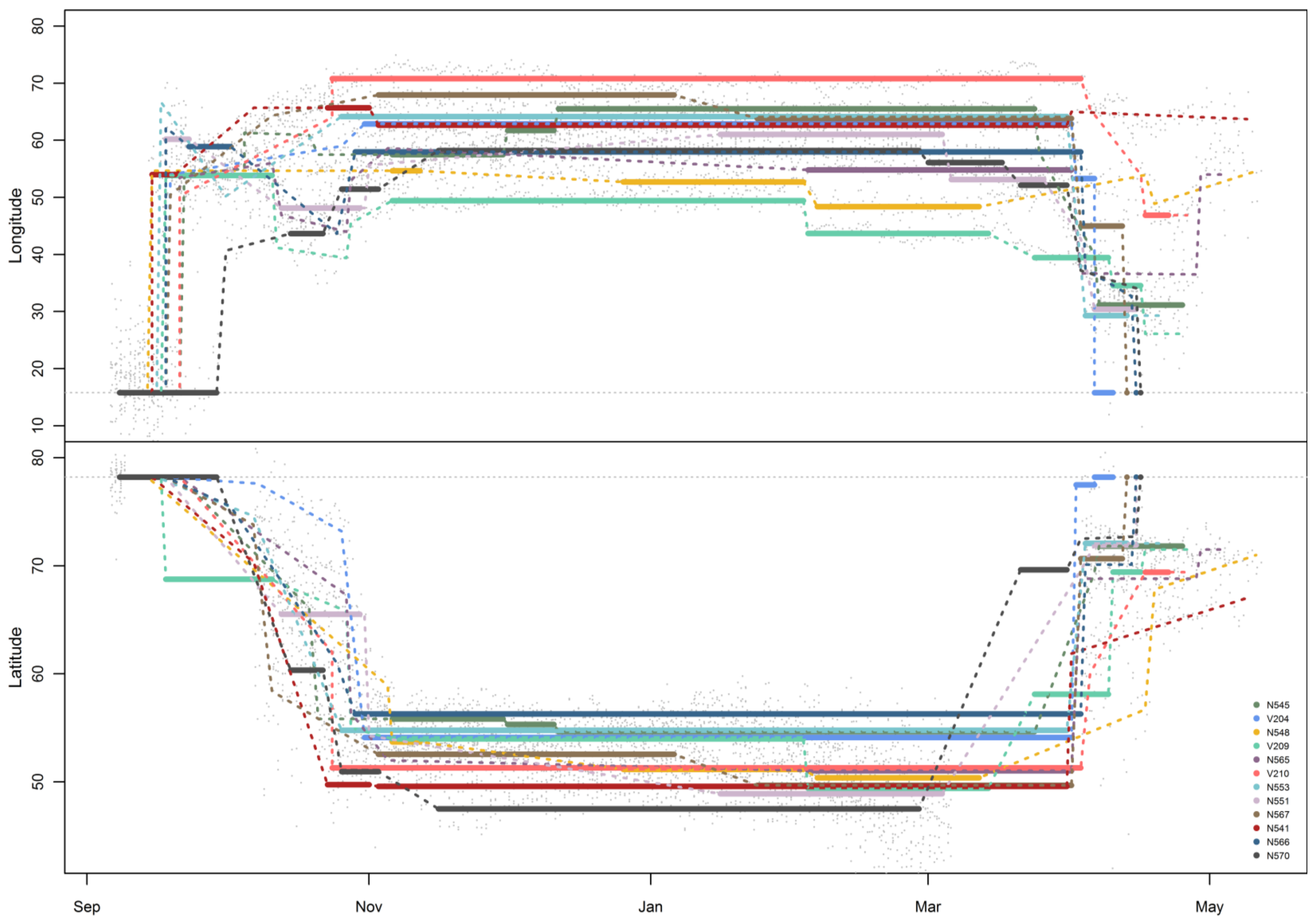

S1 Figure Migration of snow buntings from breeding grounds to breeding grounds (individuals represented by colour and logger ID). Identified stationary periods represented by solid line (—) and periods of apparent movement by a dashed line (----), these include directed migration and nomadic behaviour. Breeding site longitude and latitude indicated by grey dotted line. Estimated positions represented by grey dots, and latitudes effected by the equinox effect are excluded.
