# Supplementary material for: From Svalbard to Siberia: Passerines breeding in the High Arctic also endure the extreme cold of the Western Steppe
Source: PLoS One. 2018 Sep 5;13(9):e0202114. doi: 10.1371/journal.pone.0202114 (PMC6124700; doi:10.1371/journal.pone.0202114)
Supplement: S2 Fig — Time series of identified migratory movements, stationary periods and nomadic behaviour. Key migration events are depicted by ▼ southwards and ▲ northwards, and coloured by departure from Svalbard, autumn (red), arrival at wintering grounds (blue), departure from wintering grounds, spring (green) and where known, departure from mainland to Svalbard (yellow). Additional movements within these periods are denoted by ◆. Stationary periods are indicated by black lines (━) and apparent nomadic behaviour by grey lines. (PDF) [file pone.0202114.s002.pdf]

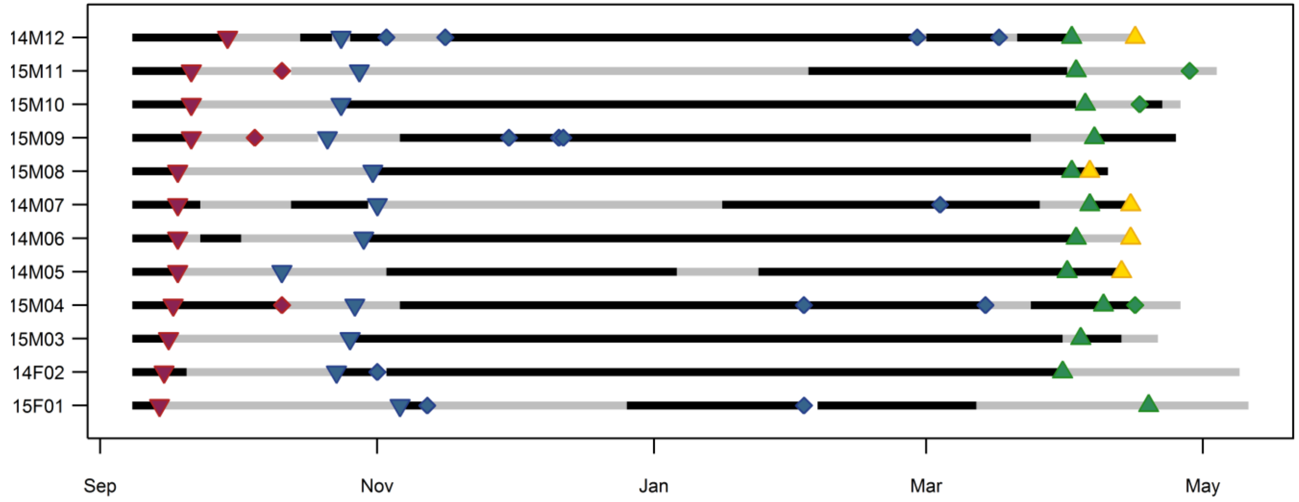

S2 Figure Time series of identified migratory movements, stationary periods and nomadic behaviour. Key migration events are depicted by ▼ southwards and ▲ northwards, and coloured by departure from Svalbard, autumn (red), arrival at wintering grounds (blue), departure from wintering grounds, spring (green) and where known, departure from mainland to Svalbard (yellow). Additional movements within these periods are denoted by ◆. Stationary periods are black lines (▬) and apparent nomadic behaviour by grey lines (▬).
