# Supplementary material for: From Svalbard to Siberia: Passerines breeding in the High Arctic also endure the extreme cold of the Western Steppe
Source: PLoS One. 2018 Sep 5;13(9):e0202114. doi: 10.1371/journal.pone.0202114 (PMC6124700; doi:10.1371/journal.pone.0202114)
Supplement: S3 Fig — Environmental variables and land use for the duration of the wintering periods, with 70% Kernel Density Estimation of wintering grounds depicted by black polygon: (a) mean daily mean surface temperature (°C), (b) absolute minimum surface temperature (°C), (c) Percentage crop cover and (d) maximum snow depth (m), note that snow cover over oceans indicates maximum extent of sea ice. (PDF) [file pone.0202114.s003.pdf]

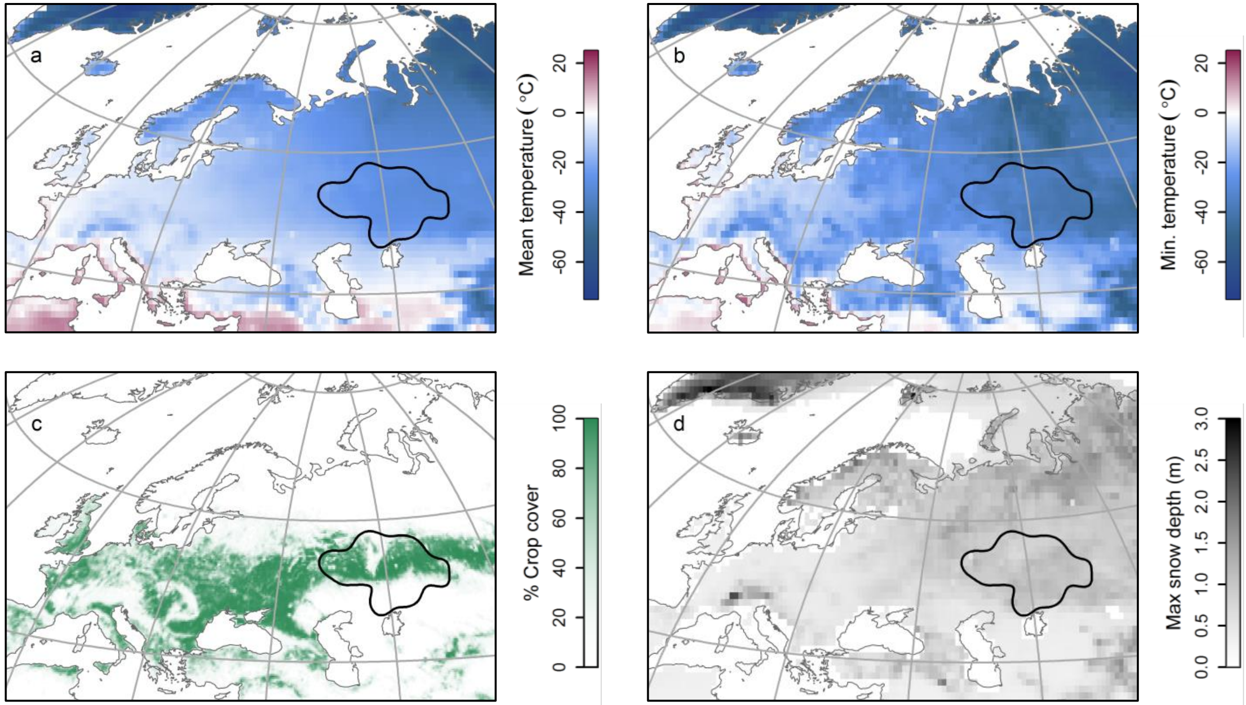

S3 Figure Environmental variables and land use for the duration of the wintering periods, with 70% Kernel Density Estimation of wintering grounds depicted by black polygon: (a) mean daily mean surface temperature (°C), (b) absolute minimum surface temperature (°C), (c) Percentage crop cover and (d) maximum snow depth (m), note that snow cover over oceans indicates maximum extent of sea ice.
