# Supplementary material for: From Svalbard to Siberia: Passerines breeding in the High Arctic also endure the extreme cold of the Western Steppe
Source: PLoS One. 2018 Sep 5;13(9):e0202114. doi: 10.1371/journal.pone.0202114 (PMC6124700; doi:10.1371/journal.pone.0202114)
Supplement: S1 Table — Tracked snow bunting deployment information and key timings of significant migration which were used to define Autumn, Winter and Spring periods. Total number of stationary periods (SP) and duration (in days) of wintering stationary periods, defined Autumn and Winter periods, and where known Spring and Breeding grounds for the 12 month period the birds were tracked. Summary statistics, where appropriate are included for day of migration and duration of periods, however the period of spring and breeding are under- and over-estimated due to lack of return dates for 7 birds due to the effect of Polar day. (PDF) [file pone.0202114.s004.pdf]

S1 Table Tracked snow bunting deployment information and key timings of significant migration which were used to define Autumn, Winter and Spring periods. Total number of stationary periods (SP) and duration (in days) of wintering stationary periods, defined Autumn and Winter periods, and where known Spring and Breeding grounds for the 12 month period the birds were tracked. Summary statistics, where appropriate are included for day of migration and duration of periods, however the period of spring and breeding are under- and over-estimated due to lack of return dates for 7 birds due to the effect of Polar day.

Year	ID	Sex	Logger	Ring no.	r	Deployment	from Svalbard	arrive Winter	arrive Spring	to Svalbard	Winter # SP	Winter SP (d)	Autumn (d)	Winter (d)	Spring (d)	Breeding (d)
2015	15F01	Female	N548	8N77713		30/06/2015	14-Sep	06-Nov	19-Apr	NA	3	80	53	165	NA	NA
	15M03	Male	N553	8N77711		30/06/2015	16-Sep	26-Oct	04-Apr	NA	1	157	40	161	NA	NA
	15M04	Male	V209	8N77722		04/07/2015	17-Sep	27-Oct	09-Apr	NA	3	144	40	165	NA	NA
	15M07	Male	V204	8N77716		02/07/2015	18-Sep	31-Oct	02-Apr	06-Apr	1	154	43	154	4	164
	15M09	Male	N545	8M97765	*	29/06/2015	21-Sep	21-Oct	07-Apr	NA	3	137	30	169	NA	NA
	15M10	Male	V210	8N77726		05/07/2015	21-Sep	24-Oct	05-Apr	NA	1	162	33	164	NA	NA
	15M11	Male	N565	8N77572	*	29/06/2015	21-Sep	28-Oct	03-Apr	NA	1	57	37	158	NA	NA
2014	14F02	Female	N541	8N77565		03/07/2014	15-Sep	23-Oct	31-Mar	NA	2	159	38	160	NA	NA
	14M05	Male	N567	8N77572		04/07/2014	18-Sep	11-Oct	01-Apr	13-Apr	2	131	23	173	12	157
	14M07	Male	N551	8M97765		03/07/2014	18-Sep	01-Nov	06-Apr	15-Apr	2	68	44	157	9	155
	14M06	Male	N566	8N77569		03/07/2014	18-Sep	29-Oct	03-Apr	15-Apr	1	157	41	157	12	155
	14M12	Male	N570	8N77588		05/07/2014	30-Sep	24-Oct	02-Apr	16-Apr	4	138	24	161	14	166
Mean							18-Sep	26-Oct	05-Apr	13-Apr	2	129	37	162	10	159
SD											1	38	9	5	4	5
Median							18-Sep	26-Oct	03-Apr	15-Apr	2	141	39	161	12	157
Min							14-Sep	11-Oct	31-Mar	06-Apr	1	57	23	154	4	155
Max							30-Sep	06-Nov	19-Apr	16-Apr	4	162	53	173	14	166
